# Benign Granular Cell Tumor of the Cecum

**DOI:** 10.7759/cureus.4074

**Published:** 2019-02-14

**Authors:** Patricia Guzman Rojas, Ernesto S Robalino Gonzaga, Vania Zayat, Jignesh Parikh

**Affiliations:** 1 Internal Medicine, University of Central Florida College of Medicine, Orlando, USA; 2 Pathology, University of Central Florida College of Medicine, Orlando, USA; 3 Pathology, Orlando Veterans Affairs Medical Center, Orlando, USA

**Keywords:** rectum, granular cell tumor

## Abstract

Granular cell tumors (GCT) are usually benign, soft tissue tumors that are mostly found in the oral cavity, skin, and subcutaneous tissue. GCTs in the gastrointestinal (GI) tract are mainly located in the esophagus.

A 63-year-old male was referred to the gastroenterology clinic for a major complaint of six months of painless rectal bleeding. Laboratory results showed mild macrocytic anemia. He denied any prior colonoscopies and hence, a lower endoscopic procedure was done. The colonoscopy showed multiple polyps, one of them located at the cecum. The cecal polyp showed polygonal cells with abundant eosinophilic infiltration and S100 stain positive. This confirmed a diagnosis of GCT.

GCTs are thought to be derived from the neural tissue (Schwann cells). This entity is usually asymptomatic; however, tumors located at the lower GI tract can present with hematochezia. Only 2% of GCTs follow a malignant course, with associated poor prognosis.

This case is being presented because of its asymptomatic nature. It is important to monitor these lesions in order to recognize early signs/symptoms concerning for malignancy.

## Introduction

Granular cell tumors (GCT) are usually benign, soft tissue tumors that can be located in any part of the body, but are mostly found in the oral cavity, skin, and subcutaneous tissues [1]. These rarely occur in the gastrointestinal (GI) tract, showing only a frequency of 4% to 6%. Furthermore, within the GI tract, the majority are located in the esophagus (65% of the cases).

## Case presentation

**Figure 1:**
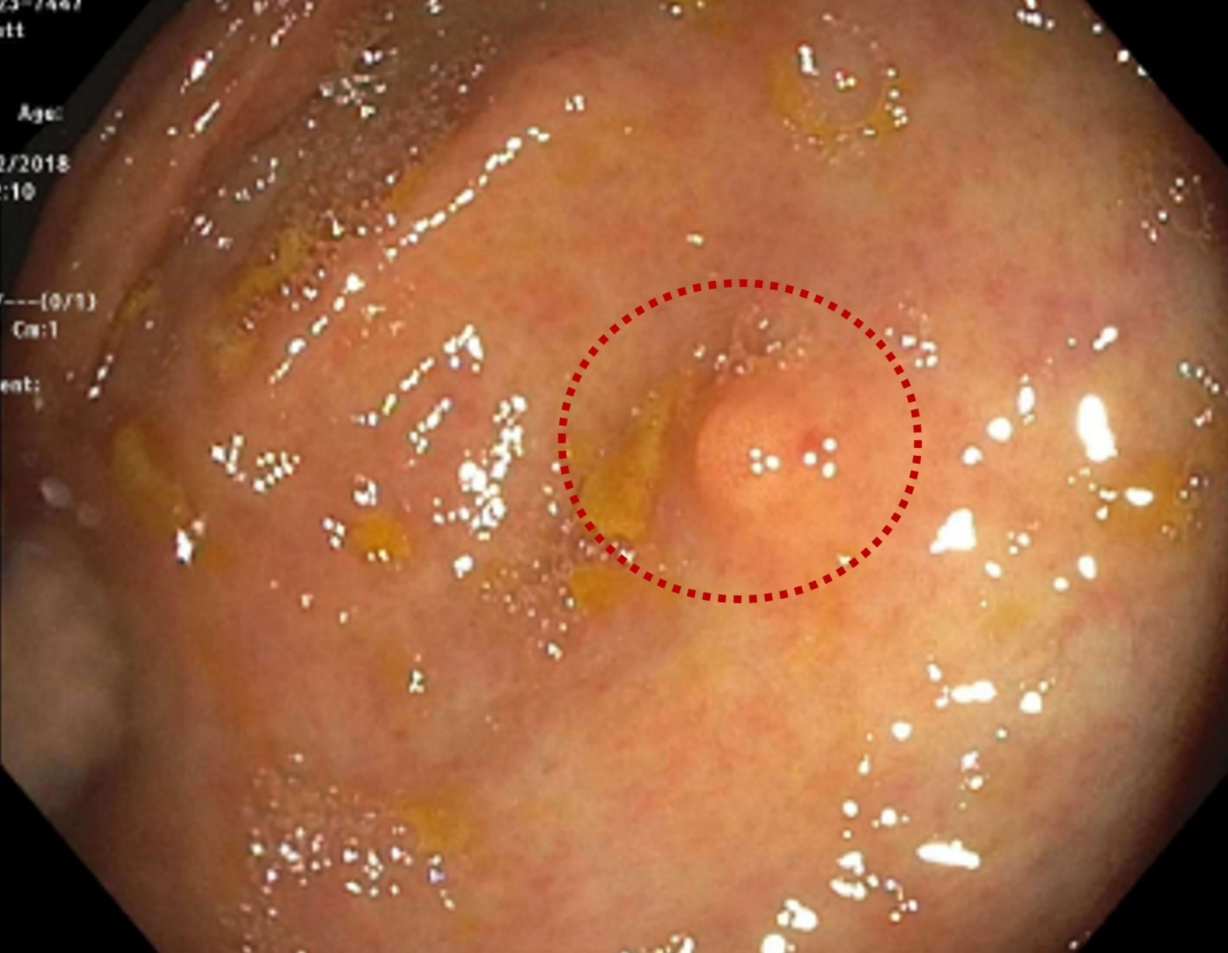
Colonoscopy showing cecum with a pale/tan sessile polyp measuring less than 5 mm, before biopsies were taken

**Figure 2:**
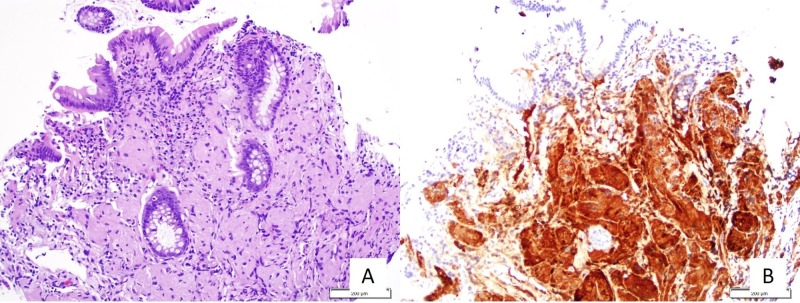
Histopathologic findings from the rectal mass: there are clusters of large cells within the lamina propria with abundant eosinophilic granular cytoplasm and small round-to-oval nuclei (A); these cells are positive for S100 immunostain (B)

A 63-year-old male with a past medical history of hypertension, human immunodeficiency virus on treatment, late latent syphilis, and chronic anemia secondary to folate deficiency was referred to the gastroenterology clinic due to painless intermittent rectal bleeding for six months. He denied any associated abdominal or rectal pain, melena, tenesmus, or mucus in his stools. Laboratory results showed a hemoglobin of 11.6 g/dL, with a mean corpuscular volume (MCV) of 105.3 fL. Since the patient did not have any prior colorectal screening procedure and given his present symptoms, a colonoscopy was indicated. The colonoscopy showed multiple polyps: one 15-mm polyp at 65 cm proximal to the anus, a 10-mm polyp at the ascending colon, a 5-mm polyp at the ileocecal valve, and a diminutive at the cecum (Figure 1). Pathologic results showed tubular adenoma features at the anal, ascending colon, and ileocecal polyps. On the other hand, the cecal polyp showed polygonal cells with abundant eosinophilic granular cytoplasm, which upon staining with S100 showed positivity and confirmed the diagnosis of a GCT (Figure 2).

## Discussion

GCTs are mainly found in patients in their fourth to sixth decades of life and have a female predominance with a 2:1 ratio [2]. These are thought to be derived from the neural tissue (Schwann cells); however, other cell lineages have been proposed as histopathogenesis [3-4]. Furthermore, Pareja et al. identified loss-of-function mutations in ATP6AP1 or ATP6AP2 genes in 72% of GCTs [5].

GCTs are frequently asymptomatic, and tumors are found incidentally on endoscopic procedures. When symptoms are present, these depend on the location of the tumor. Esophageal GCT can present as gastroesophageal reflux or dysphagia/globular sensation when the tumor is located at the cervical esophagus. Patients with small intestine tumors can present with upper gastrointestinal bleeding or abdominal pain. Colorectal GCT can present with hematochezia, abdominal pain, and change in bowel habits [1,6]. The patient presented with a polyp in the cecal area, and we believe that the intermittent rectal bleeding he experienced was a result of the multiple adenomatous polyps present along with the GCT.

Approximately 20% of all the gastrointestinal GCTs are located in the colorectal region. Usually these present as solitary tumors; however, approximately 7% to 25% can be found in aggregates [7]. The usual appearance on colonoscopy is as a small (less than 2 cm), sessile polyp with a yellow-white coloration [8]. Differential diagnosis includes lipoma, carcinoid or stromal tumors, hamartoma, and even metastatic malignancy.

The gold standard for the diagnosis, regardless of the lesion site, is based on histologic findings: there are nests of epithelioid or spindle cells, with a small round nucleus, and abundant eosinophilic granular cytoplasm. Stains are positive for S-100, CD56, CD68, SOX-10, and neuron-specific enolase [1]. Most GCTs are benign, and only less than 2% can follow a malignant course. The characteristic features that have been proposed to predict malignant potential are tumor necrosis, tumor cell spindling, pleomorphism, high nuclear to cytoplasmic ratio, large nucleoli, and increased mitotic activity [9]. If malignancy is found, this has a high propensity for metastasis, recurrence, and poor prognosis.

Since there are no established guidelines for the treatment of colorectal GCT, some physicians recommend performing endoscopic mucosal resection (EMR), endoscopic submucosal dissection (ESD), or polypectomy for tumors less than 2 cm [10]. Follow-up colonoscopies seem appropriate after resection if multiple tumors or risk of malignancy exists.

## Conclusions

Colorectal GCTs can be a diagnostic challenge due to its asymptomatic nature. Although there is a low chance for malignancy conversion, it is important to recognize this feature in order to monitor it with follow-up endoscopic procedures.
